# IL-18/IL-18R Signaling Is Dispensable for ILC Development But Constrains the Growth of ILCP/ILCs

**DOI:** 10.3389/fimmu.2022.923424

**Published:** 2022-07-08

**Authors:** Mengying Xie, Mingying Zhang, Mengyuan Dai, Shan Yue, Zhao Li, Ju Qiu, Chenqi Lu, Wei Xu

**Affiliations:** ^1^Department of Immunology, School of Basic Medical Sciences, Fudan University, Shanghai, China; ^2^Chinese Academy of Sciences (CAS) Key Laboratory of Tissue Microenvironment and Tumor, Shanghai Institutes for Biological Sciences, University of Chinese Academy of Sciences, Chinese Academy of Sciences, Shanghai, China; ^3^Department of Biostatistics and Computational Biology, State Key Laboratory of Genetic Engineering, School of Life Sciences, Fudan University, Shanghai, China

**Keywords:** innate lymphoid cell, development, cytokine, IL-18/IL-18R signaling, homeostasis

## Abstract

Innate lymphoid cells (ILCs) develop from ILC progenitors in the bone marrow. Various ILC precursors (ILCPs) with different ILC subset lineage potentials have been identified based on the expression of cell surface markers and ILC-associated key transcription factor reporter genes. This study characterized an interleukin (IL)-7Rα^+^IL-18Rα^+^ ILC progenitor population in the mouse bone marrow with multi-ILC lineage potential on the clonal level. Single-cell gene expression analysis revealed the heterogeneity of this population and identified several subpopulations with specific ILC subset-biased gene expression profiles. The role of IL-18 signaling in the regulation of IL-18Rα^+^ ILC progenitors and ILC development was further investigated using *Il18-* and *Il18r1-*deficient mice, *in vitro* differentiation assay, and adoptive transfer model. IL-18/IL-18R-mediated signal was found to not be required for early stages of ILC development. While *Il18r1^-/-^* lymphoid progenitors were able to generate all ILC subsets *in vitro* and *in vivo* like the wild-type counterpart, increased IL-18 level, as often occurred during infection or under stress, suppressed the growth of ILCP/ILC in an IL-18Ra-dependent manner *via* inhibiting proliferation and inducing apoptosis.

## Introduction

Innate lymphoid cells (ILCs) are considered the counterparts of adaptive T lymphocytes, which play a key role in rapid barrier defenses against pathogens and in tissue repair and remodeling (1–3). The ILCs were recently reclassified into five subsets, natural killer (NK) cells, ILC1s, ILC2s, ILC3s, and LTi cells, based on their development and function (4). NK cells represent the cytotoxic branch of the ILC family, while ILC1/ILC2/ILC3 belong to the helper cell branch. Most of the helper ILCs localize within tissues, especially non-lymphoid tissues including mucosal barriers, although some subsets are also present in the circulation (5–8). Aside from LTi cells that develop during fetal stage, other ILC subsets originate from common ILC progenitors downstream of common lymphoid cells (CLPs) in the bone marrow (BM) (9, 10). BM progenitors progress toward ILC lineage through gradual upregulation of ILC core transcription factors including TCF, GATA3, TOX, ID2, PLZF, and RORα. These transcription factors (and their reporter genes) together with cell surface markers have been used to identify various ILC progenitor populations (11–19). Studies on ILC progenitors revealed the heterogeneity of these populations and defined stepwise lineage restriction of ILC progenitors (15, 16, 20, 21). In the current model of ILC development, the earliest common progenitors to generate all ILC subsets including NK cells are the early ILC progenitors (EILPs; Lin^-^*Tcf^+^* 2B4^+^α4b7^+^IL-7Rα^-^Thy1^-^CD25^-^CD122^-^CXCR6^-^) that still retain residual dendritic cell (DC) potential (11, 12). Another common progenitor population is alpha lymphoid progenitors (αLPs; Lin^-^IL-7Rα^+^Flt3^-^α4b7^+^) that retains some T-cell potentials (19). These common ILC progenitors (CILCPs) then differentiate into NK progenitors (NKPs) and common helper-like ILC progenitors (CHILPs; Lin^-^*Id2*^+^Thy1^+^IL-7Rα^+^Flt3^-^α4b7^+^ CD25^-^ST2^-^) that can generate all helper ILC subsets and LTi cells (13). Within CHILPs, the committed ILC precursors (ILCPs; PLZF^+^CHILP) further lost the LTi cell potential (14), although recent studies also showed that NK cell potential is still retained in CHILPs and ILCPs, indicating that the developmental stages of these ILCPs are more complexed.

Despite increasing knowledge of the transcriptional program underlying ILC development, less is known about the extracellular signals (cytokines, growth factors, etc.) that direct ILC lineage program at early stages. Interleukin (IL)-7Rα is highly expressed on ILCs and is a key defining marker for ILCs except for mature NK cells, a reflection of its importance for ILC development and function. IL-7Rα-dependent cytokines include IL-7 and thymic stromal lymphopoietin (TSLP), both of which play a critical role in determining ILC fate and function (22, 23). IL-7 is essential for the development, proliferation, and survival of all lymphoid cell lineages (24–26). TSLP, produced by epithelial cells, enhances the type 2 immune responses of T cells and ILC2s (27, 28). Notch is another example of an extracellular factor that mediates important signals for early development of ILC. Notch 1 and Notch 2 receptors are expressed by lymphoid progenitors and ILCP (29). Notch signaling is necessary for the development ILC3 and ILC2 lineages (17, 26, 30), and Notch activity has been found to regulate the function of LTi cells in the periphery (29). However, both IL-7 and Notch are also necessary for the development of lymphoid cell lineages other than ILCs. Whether there are unique signals that determine ILCs vs. T- and B-cell fate has yet to be determined.

IL-18, a member of the IL-1 cytokine family, is known as a proinflammatory cytokine owing to its capacity to promote interferon (IFN)-γ production from T helper type 1 (Th1) cells and NK cells (31, 32). However, studies have shown that, in combination with other cytokines, IL-18 can stimulate various cell types and has pleiotropic functions. In the presence of IL-2, IL-18 induces the production of IL-3, IL-9, and IL-13 from nature killer T (NKT) and Th1 cells. Together with IL-3, IL-18 can activate mast cells and basophils to produce type 2 cytokines and effector molecules such as histamine. IL-18 has also been shown to induce IL-22 during intestinal bacterial infection (33). IL-18 signals through IL-18R that consists of the ligand-binding α chain and the coreceptor β chain then activate nuclear factor (NF)-κB and mitogen-activated protein kinase (MAPK) signaling, which leads to the expression of downstream target genes. In addition to the mature effector cells, IL-18Rα expression has also been found on BM stem/progenitor cells that mediates signaling to promote hematopoietic stem cell (HSC) quiescence during acute bacterial infection (34). IL-18Rα expression has been detected in lymphoid/ILC progenitors (15, 29, 35); however, the impact that IL-18/IL-18R signaling has on ILC development has yet to be explored.

This work characterizes an IL-18Rα-expressing ILC progenitor population in the BM. Single-cell RNA sequencing (scRNA-Seq) analysis revealed the marked heterogeneity of this population that was found to contain subsets that display various ILC subset-biased gene expression profiles. Despite high-level expression of IL-18Rα, IL-18/IL-18Rα signaling is dispensable for the generation of the population and is not required for ILC development at steady state. However, an increased level of IL-18 suppresses the expansion of IL-18Ra^+^ progenitors and their progenies in an IL-18Ra-dependent fashion *via* limiting cell proliferation and inducing apoptosis. As such, these results demonstrate a role for IL-18 in constraining ILC progenitor cell growth.

## Results

### IL-18Rα Is Expressed on Bone Marrow Innate Lymphoid Cell Progenitors and Mature Innate Lymphoid Cell Subsets

**Figure 1:**
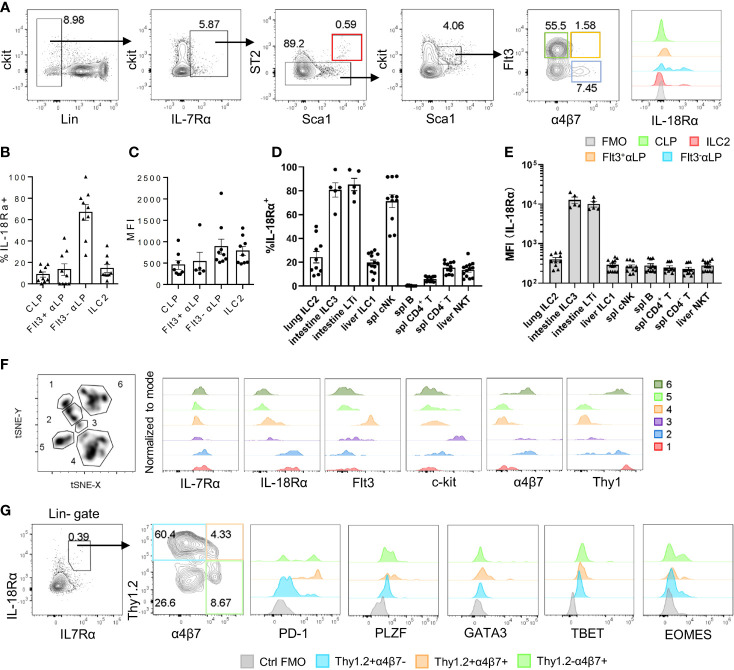
IL-18Rα is expressed on BM ILC progenitors. **(A)** The expression of IL-18Rα on BM progenitors of wild-type C57BL/6J mice was analyzed by flow cytometry. CLP, Flt3^+^αLP, Flt3^-^αLP, and ILC2 were defined as follows respectively: lin^-^IL-7Rα^+^ckit^int^Sca1^int^ST2^-^Flt3^+^α4β7^-^, lin^-^IL-7Rα^+^ckit^int^Sca1^int^ST2^-^Flt3^+^α4β7^+^, lin^-^IL-7Rα^+^ckit^int^Sca1^int^ST2^-^Flt3^-^α4β7^+^, lin^-^IL-7Rα^+^Sca1^+^ST2^+^. **(B)** Percentage of IL-18Rα^+^ cells in the progenitors in panel **(A, C)** Mean fluorescence intensity of IL-18Rα in IL-18Rα^+^ cells from CLP, Flt3^+^ αLP, Flt3^-^ αLP, and ILC2 in panel **(A)(D)** Percentage of IL-18Rα^+^ lymphocytes in peripheral tissues. **(E)** Mean fluorescence intensity of IL-18Rα in lymphocytes in peripheral tissues. **(F)** t-SNE analysis of lin-IL-7Rα^+^ cells in the BM, and expression of IL-7Rα, IL-18Rα, Flt3, ckit, α4β7, and Thy1 in 6 clusters. **(G)** Representative FACS plot showing the expression of progenitor and ILC-related markers in Thy1^+^α4β7^-^, Thy1^+^α4β7^+^, and Thy1^-^α4β7^+^ in BM IL-7Rα^+^IL-18Rα^+^ cells. Data are representative of more than three independent experiments with three mice in each group in panels **(B–E)**. The data are presented as mean ± SEM in panels **(B–E)**. Data are representative of three mice in panel **(G)**.

The expression of *Il18r1* transcript in ILC progenitors in the BM has been previously detected (15). To confirm its expression at the protein level, IL-18Rα expression was examined by flow cytometry in different BM progenitor populations. Within the lin^-^IL7Rα^+^ckit^int^ compartment, very few Flt3^+^α4b7^-^ CLPs were found to express IL-18Rα, and the frequency of IL-18Rα^+^ cells markedly increased in Flt3^-^αLP cells that contain ILC progenitors (**Figures 1A, B****)**. The expression level of IL-18Rα was also increased in this population compared to those at earlier stages (**Figure 1C**). In this study, 10%–20% of BM ILC2 was found to express IL-18Rα, with comparable levels to those in Flt3^-^αLPs. Previous work from decades past showed that conventional NK cells express IL-18R, and IL-18 in conjunction with IL-12 can not only promote the expansion and activation of NK cells but also induce the generation of memory-like NK cells (36–38). Mature ILC subsets from peripheral lymphoid organs and various non-lymphoid tissues were then examined to determine if they also express IL-18R. In agreement with published data, it was observed that over 80% of NK cells in the spleen and liver are IL-18Rα^+^ (**Supplementary Figure S1A**, **Figure 1D**). It was also found that more than half of the lung ILC2 and intestinal ILC3/LTi cells also express IL-18Rα. ILC3/LTi cells were found to express the highest level of IL-18Rα among all ILC subsets (**Supplementary Figures S1B, S1C**; **Figures 1D, E**), suggesting that IL-18 may regulate the function or homeostasis of these subsets as well. Together, these results demonstrate that IL-18Rα is expressed by lymphoid progenitors downstream of CLP in the BM and all ILC subsets in the periphery.

To better characterize the IL-18Rα-expressing lymphoid progenitor cells in the BM, total lineage-negative (Lin^-^) IL-7Rα^+^ BM cells were analyzed using FlowJo t-SNE function in an attempt to define IL-18Rα^+^ subsets within this population. This approach led to the identification of 6 clusters; clusters 1 and 2 expressed high levels of both IL-7Rα and IL-18Rα (**Figure 1F**), while cells from these two clusters differentially expressed α4b7 and Thy1. Using Thy1 and a4b7, lin^-^IL-7Rα^+^IL-18Rα^+^ cells were further divided into four subsets and analyzed to determine the expression of markers for ILCP and various ILC subsets including programmed cell death-1 (PD-1), promyelocytic leukemia zinc finger (PLZF), GATA binding protein 3 (GATA3), T-box transcription factor expressed in T cells (TBET), omesoderms (EOMES) (**Figure 1G**). None of the subsets were found to express high levels of GATA3, TBET, or EOMES, the defining transcription factors for committed ILC1, ILC2, and NK cells, thus confirming their progenitor status. The expression of PLZF and PD-1, two markers of ILCP, was detected in Thy1^+^α4b7^-^, Thy1^+^α4b7^+^, and Thy1^-^α4b7^+^ subsets, suggesting that the population of IL-18Rα^+^ cells largely overlapped with that of ILCP that was previously described (14, 39).

### IL-18Rα^+^ Bone Marrow Progenitor Cells Can Differentiate Into Multiple Innate Lymphoid Cell Subsets

**Figure 2:**
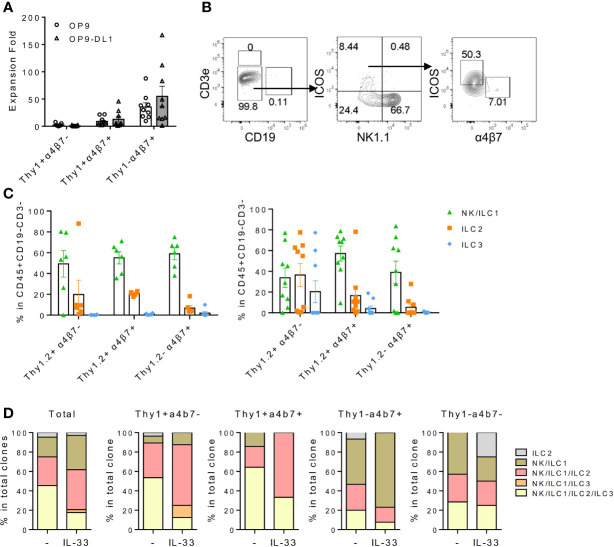
IL18Ra^+^ BM progenitor cells contain multipotent ILCPs. **(A–C)** In this study, 200~500 cells from Thy1^+^α4β7^-^, Thy1^+^α4β7^+^, and Thy1^-^α4β7^+^ subsets from the BM IL-7Rα^+^IL-18Rα^+^ population were FACS-sorted and cultured on OP9 or OP9-DL1 stromal cells with 20 ng/ml SCF and 20 ng/ml IL-7 for 12–14 days. **(A)** Expansion fold of the three subsets after culture. **(B)** Representative FACS plot showing the generated ILCs after culture. **(C)** Percentage of NK/ILC1, ILC2, and ILC3 in CD45^+^ cells generated from Thy1^+^α4β7^-^, Thy1^+^α4β7^+^, and Thy1^-^α4β7^+^ subsets cultured on OP9 (left) or on OP9-DL1 (right). **(D)** *In vitro* differentiation of index-sorted single BM lin^-^IL-18Rα^+^IL-7Rα^+^ cells on OP9-DL1 for 14–16 days. Sixty-four out of 570 clonal progenies cultured without IL-33 (indicated as “-”) and 34 out of 568 clonal progenies cultured with 20 ng/ml IL-33 (indicated as “IL-33”) were analyzed. Data are representative of three or more independent experiments with six mice in each group in panels **(A, C)** The data are presented as mean ± SEM in panels **(A, C)**.

Thy1^+^α4b7^-^, Thy1^+^α4b7^+^, and Thy1^-^α4b7^+^ subsets were next isolated from the BM Lin^-^IL-18Rα^+^IL-7Rα^+^ population and cultured on OP9 or OP9-DL1 stromal cells in the presence of IL-7 and stem cell factor (SCF). After 2 weeks, cells were harvested and counted and the generation of mature ILC subsets was analyzed by flow cytometry. We noticed that among the three populations, Thy1^-^α4b7^+^ subset produced the greatest number of cells after culture with either OP9 or OP9-DL1 stromal cells, while Thy1^+^α4b7^-^ subset expanded the least (**Figure 2A**), suggesting that Thy1^-^α4b7^+^ cells are more proliferative. T and B cells were not detected in the culture, indicating that IL-18Rα^+^ progenitor cells already lost the potential to these two lymphoid lineages (**Figures 2B, C****)**. All three subsets were observed to predominantly produce NK1.1^+^ cells (NK/ILC1) when cocultured with OP9 stromal cells, which was shown to support NK cells and ILC3 differentiation (30); less than 20% of the cells were ICOS^+^ ILC2 cells, and very few ILC3s (α4b7^+^ cells) were found in the culture. In the presence of Notch signaling, increased percentage of ILC2 was produced from the Thy1^+^α4b7^-^ subset, suggesting that this subset contains bipotent or multipotent progenitors that can give rise to ILC2 and other ILC subsets. Therefore, OP9-DL1 was used in the following *in vitro* culture experiment. To test the progenitor capacity of these cells on the single-cell level, BM lin^-^IL-7Rα^+^IL-18Rα^+^ cells were indexed-sorted and cultured on OP9-DL1 with IL-7 and SCF or additional IL-33. After 2 weeks, the growth of each well (clone) was examined under the microscope and cells from the positive wells were harvested. The generation of mature ILC subsets was assayed by fluorescence activated cel sorting (FACS) (**Supplementary Figure S1D**). The cloning frequency for these cells was found to be approximately 10% (data not shown). Among the clones that were examined, over 60% contained more than three ILC lineages, indicating that IL-18Rα^+^ cells are multipotent (**Figure 2D**), which was consistent with previous findings (35). The phenotype of the input cells from the positive clones was then reanalyzed using indexed-sorting data, and cells were divided into four subsets based on their surface expression of Thy1 and α4b7. ILC differentiation potentials of the four subsets were calculated (**Figure 2D**). Clones with multipotency were primarily from Thy1^+^α4b7^-^ and Thy1^+^α4b7^+^ cells, while Thy1^-^α4b7^+^ and Thy1^-^α4b7^-^ cells contain more ILC1P/NKPs. The addition of IL-33 increased the percentage of clones containing ILC2s in Thy1^+^α4b7^-^ and Thy1^+^α4b7^+^ cells, consistent with the bulk culture results above. Together, these data demonstrate that IL-18Rα^+^ BM progenitor cells contain multipotent ILCPs that can give rise to multiple ILC subsets.

### IL-18Rα-Expressing Bone Marrow Progenitor Population Is Heterogeneous

**Figure 3:**
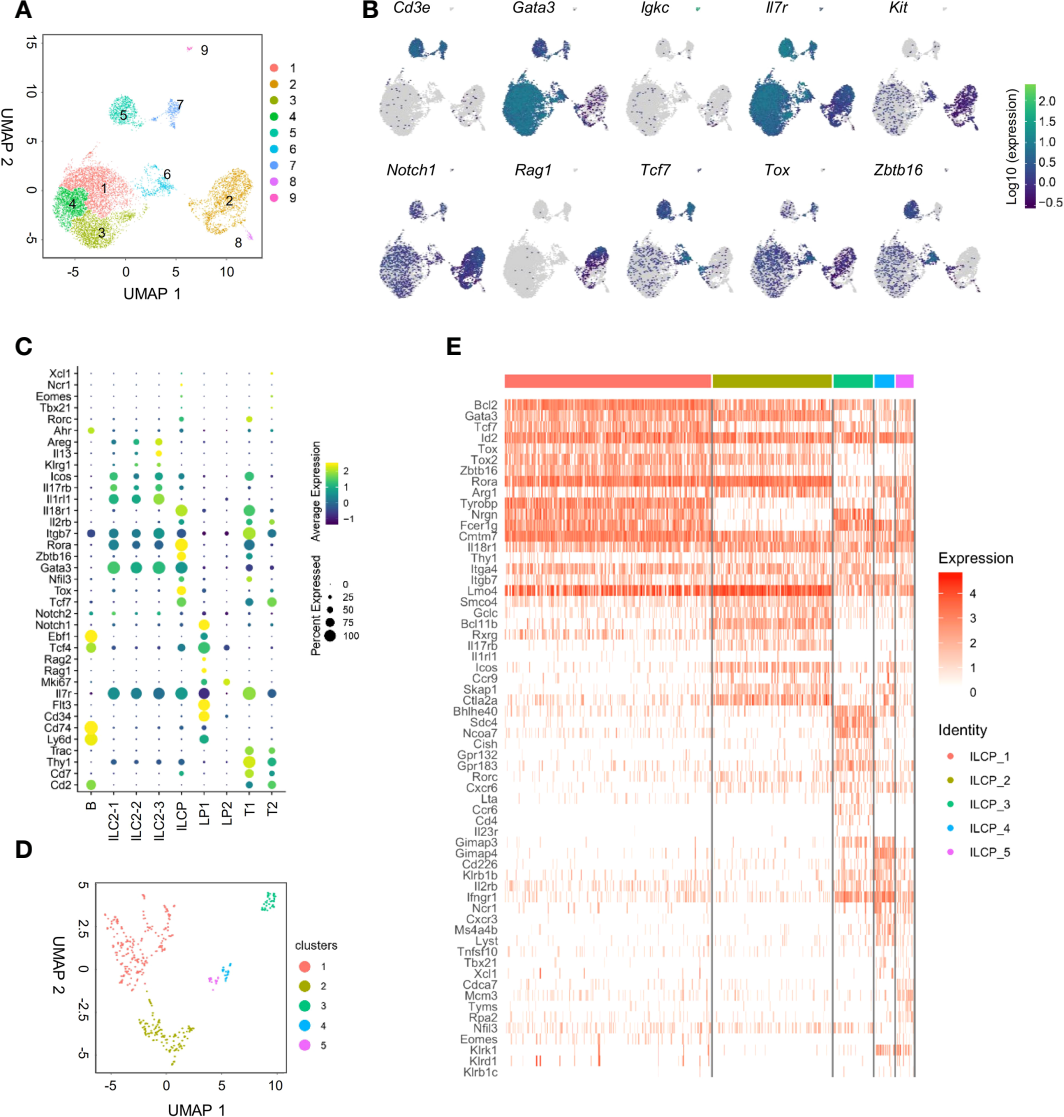
scRNA-seq analysis of adult BM lin^-^IL7Rα^+^ cells reveals the heterogeneity of the IL18Rα^+^ population. **(A)** Lin^-^IL-7Rα^+^ cells sorted from the BM of wild-type mice. UMAP plot shows the distinct clusters within 12,643 sequenced cells. **(B)** UMAP expression of lymphoid progenitor or ILC-associated genes in clusters in panel **(A, C)** Expression of candidate genes within BM lin^-^IL-7Rα^+^ cell clusters. **(D)** UMAP plot of cluster 6 from panel **(A, E)** Heatmap of selected lymphoid progenitor/ILC-associated genes by 5 clusters in panel **(D)**.

To further dissect BM IL-18Rα-expressing lymphoid progenitors on the transcriptional level in an unbiased manner, BM Lin^-^IL-7Rα^+^ cells that include lymphoid progenitor cells and committed/mature ILCs except NK cells from adult (6–8-week-old) wild-type (C57BL/6) mice were FACS-sorted for scRNA-Seq. Unsupervised clustering of the cells was performed using Leiden algorithm under UMAP, yielding 9 clusters (**Figure 3A**). All cells express *Il7r*, confirming their lymphoid identities (**Figure 3B**). Clusters 1, 3, and 4 all highly expressed *Gata3*, *Il1rl1*, and *Rora*, key markers for ILC2 (**Figures 3B, C****)**. Clusters 2 and 8 expressed higher levels of *Kit*, *Flt3*, *Cd34*, *Tcf4*, *Rag1*, and *Notch1* that are markers for lymphoid progenitors. Cluster 6 expressed *Zbtb16*, *Tcf7*, *Tox*, *Rora*, *Nfil3*, and *Gata3*, representing ILCP. Clusters 5 and 7 expressed *Cd3e*, *Cd2*, and *Trac*, while cluster 9 highly expressed *Igkc*, *Cd74*, and *Ly6d*. As this is likely due to contamination during cell purification, these three clusters were therefore omitted from further analysis. Noting that only cluster 6 highly expressed *Il18r1* and *Il18rap* (**Figure 3C**, **Supplementary Figure S2A**), we then focused on this cluster for further analysis that was found to yield 5 clusters within its population (**Figure 3D**). To transcriptionally compare these 5 subsets (ILCP1-5), these cells were subjected to unsupervised clustering and lymphoid progenitor and ILC lineage marker genes were compared across the 5 ILCP subsets (**Figure 3E**). All five ILCP subsets express *Il18r1* and *Id2* at comparable levels, which was in accordance with data from previous studies (**Supplementary Figure S2B**) (15, 35). Among the five ILCP subsets, ILCP1 had a typical PLZF^+^ILCP signature with relatively uniformed expression of *Tcf7*, *Id2*, *Rora*, *Tox*, *Tox2*, and *Zbtb16* and lower expression of *Thy1*, *Arg1*, and *Bcl11b*. Genes of differentiated ILC subsets were barely expressed in ILCP1, suggesting that this subset was the genuine multipotent ILC progenitor. ILCP2 had increased expression of *Gata3*, *Bcl11b*, *Il17rb*, *Icos*, *Ccr9*, and *Ctla2a* but minimal expression of *Il1rl1*, *Il5*, *Il13*, and *Areg*, indicating its lineage bias toward ILC2 (**Figure 3E** and data not shown). Both ILCP1 and ILCP2 express *Lmo4*, a gene previously known to be involved in precursor T-cell leukemia, which has been recently shown to be induced in ILC2 precursors and mature ILC2 (35, 40). Interestingly, ILCP3, ILCP4, and ILCP5 all upregulated genes of NK/ILC1 lineage including *Cd226*, *Klb1b*, *Il2rb*, and *Ifngr1*. Each subset also had increased expression of genes related to ILC3, ILC1, and NK cells, respectively. *Ccr6*, *Cd4*, *and Il23r* were exclusively expressed in ILCP3. *Tbx21* was expressed in some cells of the ILCP4 and ILCP5 subsets, while *Eomes* expression was found in some of the ILCP5 cells. In all five ILCP subsets, *Thy1*, *Itga4*, *and Itgb7* that encode Thy1 and α4b7 were all expressed albeit at various levels (**Supplementary Figure S2B**). All three genes were expressed at higher levels in ILCP3, which was consistent with the finding that Thy1^+^α4b7^+^ cells can generate more ILC3s *in vitro*. *Thy1* expression was lower in both ILCP1 and ILCP5 that explained our observation that Thy1^-^α4b7^+^ and Thy1^-^α4b7^-^ cells contain a mixture of ILCP and NKPs (**Figure 2F**). These results indicate that IL-18Rα^+^ ILCP is heterogeneous, containing multipotent ILCPs and ILC1/ILC2/ILC3 and NK lineage-biased progenitors.

**Figure 4:**
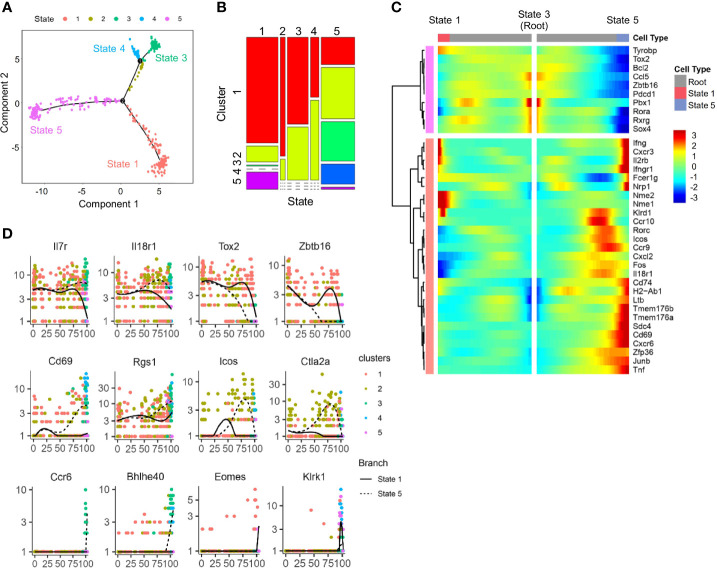
Trajectory analysis of scRNA-seq IL-18Ra^+^ BM progenitor cells. **(A)** A single cell trajectory was constructed from the dataset in **Figure 2D** (Cluster 6 cells) using Monocle package. Five distinct states were identified along the trajectory. **(B)** Distribution of five clusters identified in **Figure 2D** at five states along the trajectory in panel **(A, C)** Heatmap shows the expression profiles of lymphoid progenitor-associated and ILC-associated genes in five states along the trajectory in panel **(A, D)** Expression changes of the indicated genes related to ILCP and committed or differentiated ILCs along pseudotime from state 3 to state 1 and state 5 in panel **(A)**.

To determine the developmental relationship among ILCP subsets above, a trajectory was generated using DDR-Tree method in Monocle package. The DDRTree analysis placed individual ILCPs, regardless of cluster, into five different states (**Figure 4A**). Combining the trajectory and clustering analysis, state 3 was found to be enriched for cells of ILCP1 with higher expression of ILCP-associated genes, including *Zbtb16*, *Tox2*, and *Rora* (**Figures 4B–D**). ILCP5 cells mostly positioned in state 1 together with ILCP2, where NK-associated genes *Ifng*, *Cxcr3*, *Il2rb*, *Klrd1*, *Eomes*, and *Klrk1* were progressively upregulated (**Figures 4C, D****)**, while state 5 was enriched in ILCP2, ILCP3, and ILCP4 subsets with increased expression of ILC1-, ILC2-, and ILC3-associated genes (**Figures 4C, D****)**. In agreement with previous findings, these results support a model in which cell fate progression toward ILCP5 and subsequent NK lineage separated early from those toward ILCP3 (ILC3) and ILCP4 (ILC1).

### IL-18/IL18Rα Is Dispensable for the Development of IL18Rα+ Progenitors and Innate Lymphoid Cells

**Figure 5:**
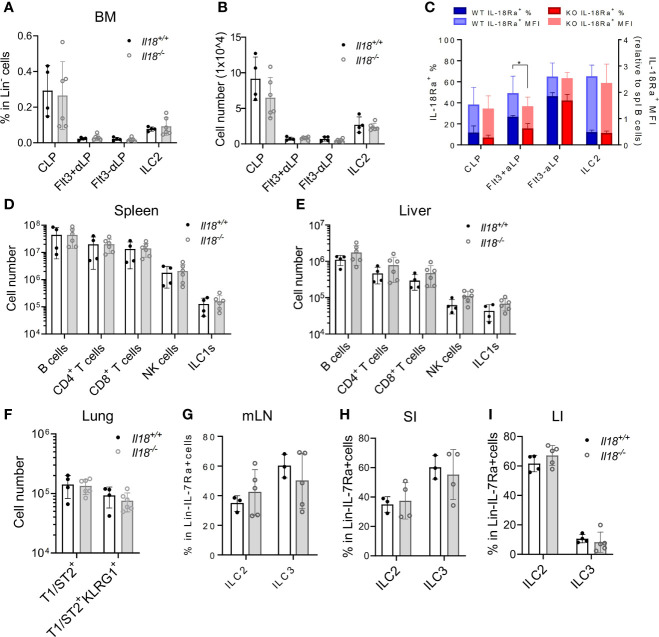
IL18 is dispensable for the generation of IL18Ra^+^ progenitors. **(A, B)** Percentage and numbers of BM progenitors and ILC2 in *Il18^+/+^* and *Il18^-/-^* mice. **(C)** Percentage of IL-18Rα^+^ cells in each population from panel **A** (presented in dark blue and dark red, respectively) and mean fluorescence intensity of IL-18Rα (presented in light blue and light red, respectively) in *Il18^+/+^* and *Il18^-/-^* mice. **(D, E)** Numbers of B cells, CD4^+^ T cells, CD8^+^ T cells, NK cells, and ILC1 cells in spleen and liver of *Il18^+/+^* and *Il18^-/-^* mice. **(F)** Number of total T1ST2^+^ ILC2 and activated T1ST2^+^KLRG1^+^ ILC2 in the lung of *Il18^+/+^* and *Il18^-/-^* mice. **(G–I)** Percentage of ILC2 and ILC3 in mesenteric lymph nodes, small intestine, and large intestine of *Il18^+/+^* and *Il18^-/-^* mice. Data are representative of three or more independent experiments with three or more mice in each group. The data are presented as mean ± SEM.

To address the question whether IL-18 signaling is required for the generation of IL-18Rα^+^ BM ILCPs, the lymphoid progenitor populations in *Il18^+/+^* and *Il18^-/-^* BM were first analyzed. No difference in the percentage or total cell number of CLP, ILCP, or BM ILC2 subsets was found (**Figures 5A, B**; **Supplementary Figure S3A**), although reduced percentage of IL-18Rα^+^ cells in the Flt3^+^αLP population, which then recovered at the Flt3^-^αLP stage, was detected (**Figure 5C**). Mature ILC subsets in the peripheral organs and mucosal tissues were next analyzed. No differences were observed in the generation of mature ILC subsets between *Il18^+/+^* and *Il18^-/-^* animals in the spleen, liver, lung, or intestinal tract (**Figures 5D–I**, **Supplementary Figures S3B–S3D**), suggesting that, at steady state, the generation of IL-18Rα^+^ ILCP and differentiated ILC subsets is not dependent on IL-18.

**Figure 6:**
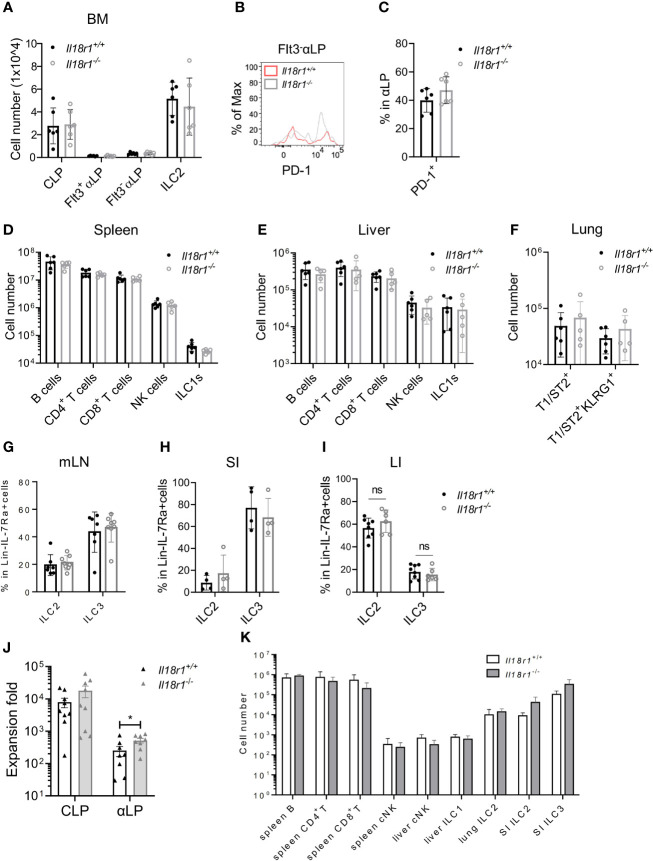
ILC development in the absence of IL18Rα signaling. **(A)** Numbers of BM progenitors and ILC2 from *Il18r1^+/+^* and *Il18r1^-/-^* mice. **(B)** Representative FACS plot showing the expression of PD-1 in Flt3^-^αLP from *Il18r1^+/+^* and *Il18r1^-/-^* BM. **(C)** Percentage of PD-1^+^ in αLP from *Il18r1^+/+^* and *Il18r1^-/-^* mice. **(D, E)** Numbers of B cells, CD4^+^ T cells, CD8^+^ T cells, NK cells, and ILC1s in the spleen and liver from *Il18r1^+/+^* and *Il18r1^-/-^* mice. **(F)** Number of total T1ST2^+^ ILC2 and activated T1ST2^+^KLRG1^+^ ILC2 in the lung of *Il18r1^+/+^* and *Il18r1^-/-^* mice. **(G–I)** Percentage of ILC2 and ILC3 in mesenteric lymph nodes, small intestine, and large intestine from *I Il18r1^+/+^* and *Il18r1^-/-^* mice. **(J)** Cell expansion of *Il18r1^+/+^* and *Il18r1^-/-^* BM CLP (50~200 cells) and αLP (100~300 cells) after culture on OP9-DL1 with 20 ng/ml SCF and 20 ng/ml IL-7 for 14 days. **(K)** Reconstitution of lymphoid cell compartment in *Rag2^-/-^Il2rγ^-/-^* mice adoptively transferred with *Il18r1^+/+^* and *Il18r1^-/-^* BM CLPs. Data are representative of two independent experiments with three mice in each group. The data are presented as mean ± SEM, analyzed by two-tailed Student’s t-test. “nc” = negative control, meaning no added IL-18 in culture system. * p < 0.05; ** p < 0.01; *** p < 0.001; **** p < 0.0001.

Next, the necessity of IL-18Rα for ILC development was investigated. BM compartments from both *Il18r1^+/+^* and *Il18r1^-/-^* animals were examined, and the numbers of CLP, αLP, or ILC2 were found to be comparable (**Figure 6A**, **Supplementary Figure S4A**). Since IL-18Rα could not be detected in *Il18r1^-/-^* animals (**Supplementary Figure S4B**), tests were made for other surface markers. PD-1 and IL-18Rα were found to be coexpressed by the Lin^-^IL-7Rα^+^IL-18Rα^+^ population (**Supplementary Figure S4C**); therefore, PD-1 was used as a surrogate marker to determine the IL-18Rα^+^ ILCP in *Il18r1^-/-^* animals. No major difference in the percentage of PD-1^+^ αLP in the BM was observed (**Figures 6B, C****)**, suggesting that the *Il18r1* deficiency did not impair the generation of this ILCP population. No loss of differentiated ILC subsets was detected in the periphery including spleen, liver, lung, and intestinal lymphoid tissues (**Figures 6D–I**). To exclude the non-hematopoietic effect caused by *Il18r1* deficiency, *Il18r1^-/-^* and *Il18r1^+/+^* CLPs and αLP were purified and had their ILC differentiation potential examined *in vitro*. After 14 days of coculture with OP9-DL1 stromal cells, the generation of ILC subsets was determined by FACS analysis. We noticed that *Il18r1^-/-^* and *Il18r1^+/+^* CLPs expanded in culture to a similar extent, while *Il18r1^-/-^* αLP produced more cells compared to *Il18r1^+/+^* counterpart after culture (**Figure 6J**). Despite that, the production of NK/ILC1 and ILC2 was also comparable between *Il18r1^+/+^* and *Il18r1^-/-^* seeded cells (**Supplementary Figures S5A, S5B**). To test the intrinsic ILC differentiation capacity of *Il18r1^-/-^* BM progenitors *in vivo*, CLPs from *Il18r1^+/+^* and *Il18r1^-/-^* BM were FACS-sorted and transferred into sublethally irradiated *Rag1^-/-^γc^-/-^* recipients. The generation of mature ILC subsets was examined by FACS 5 weeks posttransplantation. Both populations generated mature ILC subsets, including NK, ILC1, ILC2, and ILC3 in various organs (**Figure 6K**, **Supplementary Figures S5C–S5E**). The number of T and B cells in the spleen was also comparable. Together, these data indicated that the formation of IL-18Rα^+^ ILCP and differentiation of downstream ILC subsets do not require IL-18R signaling.

### IL-18 Suppresses the Growth of Innate Lymphoid Cell Precursor/Innate Lymphoid Cells

**Figure 7:**
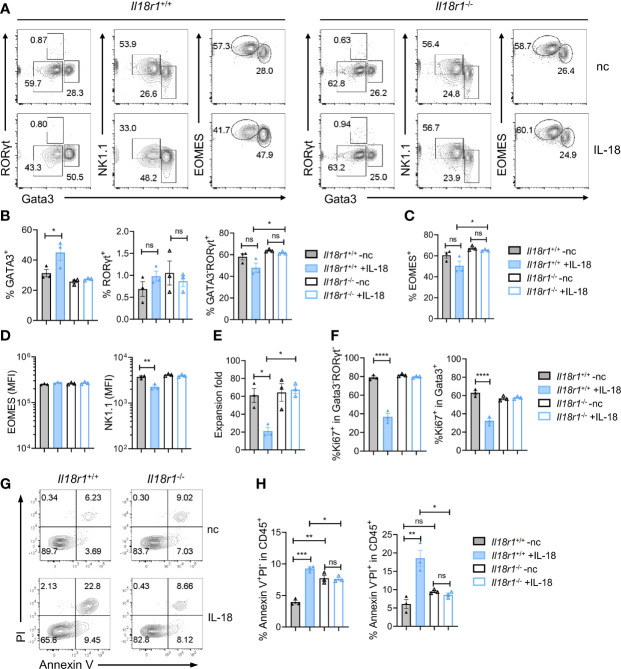
IL-18 suppresses the growth of ILCP/ILCs. Lin^-^ST2^-^PD1^+^IL-18Rα^+^ cells (350~700 cells) were FACS-sorted from *Il18r1^+/+^* and *Il18r1^-/-^* BM and cultured on OP9-DL1 in the presence of SCF (20 ng/ml) and IL-7 (20 ng/ml) with or without IL-18 for 7 days. **(A)** Representative FACS plot showing NK/ILC1, ILC2, and ILC3 generated from the culture. **(B, C)** Percentage of the NK/ILC1, ILC2, and ILC3 in CD45^+^ cells from the culture. **(D)** Mean fluorescence intensity of EOMES and NK1.1 in GATA3^-^RORγt^-^ cells. **(E)** Expansion fold of the cultured cells. **(F)** Percentage of Ki67^+^ cells in the indicated cell populations. **(G–I)** Representative FACS plot and statistical analysis showing the percentage of Annexin V^+^PI^-^ **(H)** and Annexin V^+^PI^+^ **(I)** cells in CD45^+^ cells from the culture. Data are representative of two independent experiments with three mice in each group. The data were presented as mean ± SEM, analyzed by two-tailed Student’s t-test. * p < 0.05; ** p < 0.01; *** p < 0.001; **** p < 0.0001.

The level of IL-18 increases during systemic bacterial infection or under stress conditions, which can enhance IFN-γ production (31). Recent findings have demonstrated that IL-18R signaling is required for promoting HSC quiescence during acute bacterial infection. IL-18 in combination with IL-7 was found to promote the expansion of early T-cell progenitors from the thymus and hematopoietic stem cells and lymphoid progenitor cells from the BM in part *via* upregulation of c-kit and IL-7Rα on the cell surface (41). To test the role of IL-18 in regulating ILCP homeostasis, Lin^-^ST2^-^PD1^+^IL-7Rα^+^ progenitors were purified from *Il18r1^+/+^* and *Il18r1^-/-^* BM and cultured on OP9-DL1 in the absence or presence of IL-18 in addition to IL-7 and SCF. The cultured cells were analyzed 7 days later by FACS (**Figure 7A**). Under the conventional culture condition, the percentages of ILC2, ILC3, and ILC1/NK cells generated from *Il18r1^+/+^* and *Il18r1^-/-^* progenitors were comparable. However, an increased percentage of ILC2 cells and a reduced percentage of NK/ILC1 were observed from *Il18r1^+/+^* progenitors cultured with IL-18, which was not found in *Il18r1^-/-^* cells under the same culture condition (**Figures 7B, C****)**. Interestingly, the expression of NK1.1, one of the surface markers for NK/ILC1, was reduced in *Il18r1^+/+^* cells cultured with IL-18 (**Figure 7D**). We speculated that IL-18 may downregulate the expression of NK1.1 on NK/ILC1 cells rather than suppress the differentiation of NK/ILC1 from IL-18Ra^+^ progenitors, as the expression of EOMES was similar between *Il18r1^+/+^* and *Il18r1^-/-^* regardless of their culture condition (**Figure 7D**). Further analysis revealed that *Il18r1^+/+^* cell expansion was much reduced when treated with IL-18, accompanied by decreased proliferation in all ILC subsets (**Figures 7E, F****)**. This growth-suppressive effect was IL-18R dependent, as the proliferation of *Il18r1^-/-^* cells was not affected by addition of IL-18. In addition to reduced proliferation, increased apoptosis and cell death were also observed in *Il18r1^+/+^* cells treated with IL-18 (**Figures 7G, H****)**. These results together demonstrated that ectopic IL-18 can suppress the growth ILCP and ILCs by inhibiting cell proliferation and promoting apoptosis.

## Discussion

In this study, we characterized a BM ILC progenitor population that expresses IL-18Rα and the role of IL-18/IL-18R in ILC development. By scRNA-seq analysis and clonal assay, this Lin^-^IL-7Rα^+^IL-18Rα^+^ population was found to be transcriptionally heterogeneous and contained several subsets with multi- or uni-ILC lineage differentiation potential. The BM IL-18Rα^+^ ILC progenitors were present in both *Il18^-/-^* and *Il18r1^-/-^* animals in normal numbers, and ILC differentiation in the periphery was also undisturbed. These findings suggest that IL-18/IL-18Rα signaling is not required for the generation of ILCP or ILC development at steady state. Nevertheless, ectopic IL-18, as seen in some inflammatory diseases, can inhibit ILCP/ILC expansion and induce apoptosis.

The expression of *Il18r1* in the BM Lin^-^ cells has been previously studied *via* scRNA-seq of Arg1^+^ cells from the BM (42). These Lin^-^Arg1^+^ (Yarg^+^) cells were defined as BM ILC2 in the study, and the differential expression of *Il18r1* within the Arg1^+^ ILC2 was attributed to the transcriptional heterogeneity of ILC2s. In the scRNA-seq data produced by our study, *Arg1* expression was detected in ILC2 and ILCP (clusters 1, 3, 4, and 6), while *Il18r1* was exclusively expressed in ILCP (cluster 6) and only was sparsely expressed in ILC2-1 cells (cluster 1) (data not shown). Within the ILCP, the expression of *Arg1* was not homogeneous and displayed a gradual increase from ILCP1 subset to ILCP2 subset, which represents an ILC2-biased progenitor population. Therefore, the expression of *Il18r1* in the BM is considered to mark a progenitor population rather than differentiated ILC2. Recently, two similar IL-18Rα^+^ ILC progenitor populations were reported in mice (35, 43). Using an RORα fate-mapping model, Ghaedi et al. (43) identified a Lin^-^RORα(YFP)^+^Thy1^+^IL7Rα^+^IL18Rα^+^ST2^-^ population in the mouse adult and neonatal lung. This population had an expression profile that resembles BM ILCP (18) and can give rise to multiple ILC lineages but fewer NK cells. This population was thereby defined as lung ILCPs. Interestingly, the gene expression profile of these lung ILCPs was somewhat similar to that of ILCP2 subsets in our study. Both express high levels of *Gata3* and *Rora*, a reduced level of *Tcf7* and *Zbtb16* expression compared to the true ILCP (ILCP1 in our analysis), little expression of *Il1rl1*, but upregulation of *Thy1*, *Il17rb*, and *Icos*, suggesting that these BM ILC2-biased ILCP2 could potentially serve as one source of lung ILCP from the BM. By scRNA-seq analysis of Lin^-^IL7Ra^+^ “pan-ILC” in the uninfected lung tissue, Zeis et al. (35) also found similar IL-18Ra^+^ tissue-resident ILC progenitors that primarily give rise to ILC2. In the same study, a BM IL-18Rα^+^ICOS^+^ ILC population that contained ILCP with ILC2 bias was also analyzed. In agreement with their finding, our study also detected one similar population within our BM IL-18Rα+ ILCP, namely, ILCP2 with expression of *Icos* and upregulation of ILC2 genes.

The scRNA-seq data from our study clearly shows the heterogeneity of the BM IL-18Ra^+^ ILCPs containing genuine PLZF^+^ILCPs as well as ILC2, ILC3/ILC1/NK-biased progenitors (**Figure 3E**), which resembled the *Id2^+^* ILCP identified previously (15). In that study, the *Il18r1* transcript was also found to be expressed in both *Id2^+^* ILCP1 and *Id2^+^* ILCP2 regardless of their expression of PLZF or *Bcl11b*. In the current study, all IL-18Ra^+^ ILCP subsets express *Id2*, suggesting that these ILCPs from the two studies may overlap to a great extent. Among the IL-18Ra^+^ ILCP subsets, ILCP2 was relatively distinguished from other subsets even though it also expresses *Rorc* and *Cxcr6* at comparable levels to those in the ILCP3-5 subsets, as well as *Ifngr1*, albeit at lower levels compared to those in other subsets. This gene expression profile is consistent with its multilineage differentiation potential in the clonal assay. ILCP3 also displayed a distinct gene expression profile including the upregulation of *Lta*, *Ccr6*, *Cd4*, and *Il23r*, although genes of ILC1/NK lineages such as *Il2rb*, *Cd226*, and *Ifngr1* were also induced. Interestingly, the expression level of *Bhlhe40*, a basic helix-loop-helix transcription factor, was markedly increased in the ILCP3 subset. The expression of *Bhlhe40* can be induced by a variety of environmental stimuli including retinoic acid (RA) (44), and it has been reported to play multiple roles in regulating the immune cell function (45, 46). Whether it also regulates ILC development and function requires further investigation. The gene expression profiles of the ILCP4 and ILCP5 subsets are much more similar. *Tbx21* and *Xcl1* expression was low but present in both populations, while *Nfil3* and *Eomes* were mainly expressed by ILCP5 cells that, even at low levels, indicates its NK-biased identity. These data were consistent with another published BM ILC scRNA-seq data (16) in which BM Lin^-^Id2^+^ cells were examined. However, this population included both IL-7Ra^+^ and IL-7Ra^-^ cells that presumably would contain further downstream NK/ILC1 progenitors with higher expression of NK/ILC1 lineage genes that are not seen in the data from this study.

The expression of IL-18Rα on ILCP suggests that it may be functionally important. However, no impairment in the formation of ILCP or ILC development was observed in the absence of IL-18 or IL-18Rα at steady state condition. *In vivo* transfer of *Il-18r1^+/+^* or *Il-18r1^-/^*^-^ CLPs into *Rag1^-/-^γc^-/-^* recipients resulted in comparable reconstitution of ILCs and T/B lymphocytes, suggesting that IL-18 is not essential for the development of ILCP. In contrast to the previous finding that IL-18 induced the expansion of early T cell precursor (ETP) and BM progenitors, our study found that IL-18 had a suppressive effect on IL-18Rα^+^ ILCPs. The addition of IL-18 inhibited the expansion of ILCP and its progenies due to reduced proliferation and increased apoptosis, and the inhibition is IL-18Ra dependent. This discrepancy might be due to the differences in the intrinsic properties of these progenitor cells, as the growth inhibitory effect of IL-18 was also observed in another study that found that IL-18 functions to regulate the quiescence of ST-HSCs (47). The effect was more prominent under the stress condition in a transplantation model. Interestingly, the suppressive effect of IL-18 has also been reported in a non-hematopoietic system in which IL-18 may inhibit cell differentiation or induce cell death in neural progenitor cell culture (48). Therefore, it will be of interest to explore whether IL-18 plays a role in regulating ILCP/ILCs under specific conditions of infection or disease in the future.

## Materials and Methods

### Mice

C57BL/6J mice were purchased from Shanghai Lab Animal Research Centre, while C57BL/6J *Il18r1^-/-^* mice were purchased from Cyagen Biosciences. *Il18^-/-^* mice were a generous gift from Professor Guangxun Meng of the Institute Pasteur of Shanghai, Chinese Academy of Sciences. *Rag1^/-^ Il2rg^-/-^* mice were generously gifted from Dr. Ju Qiu, Shanghai Institutes for Biological Sciences, Chinese Academy of Sciences. All mice were bred in a specific pathogen-free environment. All experiments were carried out according to the Council on Animal Care at Fudan University. The mice used for BM sorting were aged 5–7 weeks old. The mice used for flow cytometry analysis were 6–8 weeks old. The mice used for BM transplantation were 9 weeks old.

### Cell Line and Cell Culture

OP9 and OP9-DL1 stromal cells were cultured in RPMI 1640 (Solarbio, Cat#31800) containing 2.05 mM L-glutamine, 23.8 mM sodium bicarbonate, 25 mM N-2-hydroxyethylpiperazine-N-2-ethane sulfonic acid (HEPES), 0.188 mM penicillin G sodium salt, and 0.0686 mM streptomycin sulfate, supplemented with 10% fetal bovine serum (FBS). For coculture experiments, OP9 or OP9-DL1 stromal cells were preseeded in a 96-well plate at a density of 1,000 cells/well 1 day earlier. Sorted progenitors were plated on the stromal cells and cultured with RPMI 1640 or Opti-MEM (GibcoTM, Cat#31985070) with 10% FBS, 50 μg/ml gentamycin sulfate (Sigma-Aldrich, Cat#345814), and 55 μM 2-mercaptoethanol (Sigma-Aldrich, Cat#M7522). SCF (20 ng/ml, Novus, Cat#NBP2-35150), Flt3L (20 ng/ml, Novus, Cat#427-FL-005), IL-7 (20 ng/ml, Novus, Cat#NBP2-35136), IL-33 (20 ng/ml, Novus, Cat#NBP2-35124), or IL-18 (20 ng/ml, Sino Biological, Cat#50073-MNCE) was added to the medium when specified. Half of the medium was removed and was replaced by fresh medium every 4–5 days. All cells were cultured at 37°C and 5% CO_2_.

### Cell Preparation

For the BM, femora and tibiae were crushed in 3 ml FACS buffer [1× PBS (Solarbio, Cat#P1020) containing 2% FBS]. Red blood cells were removed by incubating in RBC Lysis buffer (TONBO, TNB-4300), and the cells were then resuspended in FACS buffer. For the lung, the excised lung was cut into pieces and digested in 6 ml RPMI 1640 (Solarbio, Cat#31800) containing 2% FBS, 1 mg/ml collagenase IV (Sigma-Aldrich, Cat#C5138), and 50 U/ml DNaseI (Sigma-Aldrich, Cat#260913) on a 40-μm strainer in a 6-well plate at 37°C for 45 min. The cells were ground through the 40-μm strainer, and the red blood cells were removed by incubating in RBC Lysis buffer (TONBO, Cat#TNB-4300). Spleens were ground through a 70-μm nylon mesh, and the red blood cells were removed by incubating in RBC Lysis buffer (TONBO, Cat#TNB-4300). Livers were chopped in pieces and ground, the cells were then passed through a 70-μm strainer, the red blood cells were removed by incubating in RBC Lysis buffer (TONBO, Cat#TNB-4300), and the lymphocytes were isolated by 40%:80% gradient of Percoll (GE Healthcare, Cat#17-0891-01) at 1,258×g with 0 accel/brake for 25 min. For lamina propria cells in the intestinal tract, Peyers’ patches were removed from the small intestine (SI) and colon, and their contents were washed out using FACS buffer. The intestinal canal was then first cut longitudinally, then into 1-cm-long segments. The segments were stirred at 180 rpm in 30 ml Hank´s Balanced Salt Solution without calcium and magnesium (D-HBSS) (Solarbio, Cat#H1045) complemented with 5 mM Ethylene Diamine Tetraacetic Acid (EDTA) (Sangon Biotech, Cat#B540625) and 0.05 mM dithiothreitol (DTT) (BBI, Cat#A620058) at 37°C for 30 min to wipe off the mucus on the inside lining of the tract. The segments were then minced into pieces and digested at 37°C for 30 min in 6 ml RPMI 1640 containing 2% FBS, 1 mg/ml collagenase (Sigma-Aldrich, Cat#C5138), 50 U/ml DNase I (Sigma-Aldrich, Cat#260913), and 0.05 mg/ml Dispase II (Sigma-Aldrich, Cat#D4693) on a 100-μm cell strainer in a 6-well plate. The digestive solution was passed through a 100-μm cell strainer. The lymphocytes were isolated by 40%:80% gradient of Percoll (GE Healthcare, Cat#17-0891-01) at 1,258×g with 0 accel/brake for 25 min.

### Antibodies and Flow Cytometry

Detailed information on antibodies was listed in **Supplementary Table 1**.

Ghost Dye Violet 510 (TONBO, Cat#13-0870) was used to label the dead cells, and CD16/CD32 (2.4G2) was used to block the Fc receptors (FcRs) on the cell surface before staining. Lineage cells were labeled by a cocktail of antibodies [B220 (RA3-6B2), CD3e (145-2C11), CD11b (M1/70), CD11c (N418), Ly6G/6C (RB6-8C5), TCRβ (H57-597), γδTCR (GL-3), NK1.1 (PK136), and Ter119 (TER-119)]. Cell suspensions from the BM were incubated with fluorescein-conjugated antibodies against lineage (Streptavidin Super Bright 780, Invitrogen, Cat#78-4317-82), ckit (ACK2), IL-7Rα (A7R34), Sca1 (D7), ST2 (DJ8), Flt3 (A2F10), and α4β7 (DATK32) to detect common lymphoid progenitors (CLPs), innate lymphoid cell progenitors (αLP), and ILC2 progenitors (ILC2Ps). Cells from the lung were incubated with fluorescein-conjugated antibodies against lineage (Streptavidin Super Bright 780, Invitrogen, Cat#78-4317-82), CD45 (30-F11), Thy1.2 (53-2.1), ST2 (RMST2-2), and KLRG1 (2F1) to detect ILC2. Cells from the spleen and liver were incubated with fluorescein-conjugated antibodies against CD45 (30-F11), CD19 (ebio1D3), CD3 (17A2), NK1.1 (PK136), CD4 (GK1.5), CD8a (53-6.7), NKp46 (29A1.4), and CD49b (DX5) to detect B cells, T cells, NK cells, and ILC1. Cells from the intestinal tract (small intestine and colon) were first labeled with biotin-conjugated CD3e (145-2C11) and CD19 (ebio1D3), then incubated with a mixture of fluorescein-conjugated antibodies against IL-7Rα (A7R34), Thy1.2 (53-2.1), NKp46 (29A1.4), and CCR6 (140706) to detect NKp46^+^ ILC3 and LTi-like ILC3. For intracellular staining of transcription factors, cells were fixed and permeabilized using Transcription Factor Fix/Perm Concentrate (4×) (TNB-1020-L050) and Transcription Factor Fix/Perm Diluent (1×) (TNB-1022-L160), then incubated in Flow Cytometry Perm Buffer (TNB-1213-L150) with fluorescein-conjugated antibodies against Gata3 (L50-8233) and RORγt (AFKJS-9). The cell suspensions were analyzed using BD FACS CelestaTM (BD Biosciences) and BD FACSDivaTM Software (BD Biosciences).

For cell sorting, lineage [B220 (RA3-6B2), CD3e (145-2C11), CD11b (M1/70), Ly6G/6C (RB6-8C5), Ter119 (TER-119)]-positive cells in the BM were labeled with Miltenyi Anti-Biotin MicroBeads (Miltenyi Biotec, Cat#130-090-485) and applied to the Miltenyi LS Columns (Miltenyi Biotec, Cat#130-0420401) in a magnetic field. Lineage-negative cells were washed out with FACS buffer according to the manual. The enriched lineage-negative cells were incubated with fluorescein-labeled antibodies against lineage (Streptavidin Percp-eFluor 710, Invitrogen, Cat#46-4317-82), ckit (ACK2), IL-7Rα (A7R34), Sca1 (D7), ST2 (DJ8), Flt3 (A2F10), and α4β7 (DATK32) for CLP and αLP, lineage (Streptavidin Percp-eFluor 710, Invitrogen, Cat#46-4317-82), ckit (ACK2), IL-7Rα (A7R34), IL18Rα (P3TUNYA), Thy1.2 (53-2.1), and α4β7 (DATK32) for IL-7Rα^+^IL-18Rα^+^ cells. The progenitors were then purified using BD FACSAria II (BD Biosciences). For index sorting, BM cells were stained with fluorescein-labeled antibodies against lineage (Streptavidin Percp-eFluor 710, Invitrogen, Cat#46-4317-82), IL-7Rα (A7R34), IL18Rα (P3TUNYA), Thy1.2 (53-2.1) and α4β7 (DATK32), and ST2 (DJ8) and ICOS (7E.17G9), then IL-7Rα^+^IL-18Rα^+^ single cells were directly sorted into a 96-well plate preseeded with OP9-DL1 stromal cells using MA900 Multi-Application Cell Sorter (Sony Biotechnology).

### *In Vivo* Adoptive Transfer

FACS-sorted BM CLPs from *Il18r^+/+^* and *Il18r1^-/-^* mice were washed, resuspended with 1× PBS, and then were injected intravenously into sublethally irradiated (5.5 Gy) *Rag2^-/-^Il2rg^-/-^* mice through the tail. In this study, 1 mg/ml neomycin (BBI, A610366) was added into the drinking water during the first 3 weeks to avoid infection. The recipient mice were sacrificed 5 weeks after implantation, and the various organs and tissues were analyzed.

### Single-Cell RNA Sequencing and Bioinformatic Analysis

BM lin^-^IL-7Rα^+^ cells of wild-type mice were FACS-sorted and loaded on the Chromium Single Cell Controller (10× Genomics). An scRNA-Seq library of mixed mouse BM samples was generated by using the 10× Chromium platform 3′ v3 preparation kit (10× Genomics) following the manufacturer’s recommendations. CellRanger v6.0.0 (10× Genomics) was then utilized to align reads onto Mus musculus UCSC version mm10 (mm10) reference genome and generated a gene-cell count matrix. Seurat v4.0.5 packages and R v4.0.3 were used to perform quality control, data preprocessing, dimensionality reduction, clustering, and visualization of the scRNA-Seq data. These low-quality cells containing less than 1,000 identified genes or more than 20% of reads arising from mitochondrial genes were removed, while the doublets were filtered by scDblFinder v1.4.0 package. After normalizing and scaling the gene-cell matrix, dimensionality reduction and visualization were performed using Principal Components Analysis (PCA) and Uniform Manifold Approximation and Projection (UMAP). The cell clusters were divided under the Leiden algorithm, and the highly expressing markers of each cluster were identified by FindALLMarkers function in Seurat package. The cell trajectory analysis was completed by monocle v2.18.0 packages, and the cell states were generated under the dimensionality reduction method of DDRTree. These genes associated with trajectory analysis were then identified and demonstrated by monocle packages. Publicly available datasets were analyzed in this study. These data can be found here: https://www.biosino.org/node/review/detail/OEV000317?code=UVSFETX5

### Quantification and Statistical Analysis

Samples analyzed *via* flow cytometry were visualized and quantified with FlowJo v10 software (Tree Star). Quantified data were analyzed with GraphPad Prism 8 software (GraphPad Software, Inc.) by two-tailed unpaired Student’s t-test. The compared data were presented as mean ± SEM.

## Data Availability Statement

Publicly available datasets were analyzed in this study. This data can be found here:https://www.biosino.org/node/review/detail/OEV000317?code=UVSFETX5.

## Ethics Statement

The animal study was reviewed and approved by Council on Animal Care, Fudan University.

## Author Contributions

WX and MX designed the research. MX, MZ, and MD performed the experiments and analyzed data. CL performed the bioinformatics analyses of scRNA-seq data. SY, ZL and JQ helped with the experiments. WX supervised the research and wrote the paper. All authors contributed to the article and approved the submitted version.

## Funding

This work was supported by the National Natural Science Foundation of China (grants 31770958 and 81701543).

## Conflict of Interest

The authors declare that the research was conducted in the absence of any commercial or financial relationships that could be construed as a potential conflict of interest.

## Publisher’s Note

All claims expressed in this article are solely those of the authors and do not necessarily represent those of their affiliated organizations, or those of the publisher, the editors and the reviewers. Any product that may be evaluated in this article, or claim that may be made by its manufacturer, is not guaranteed or endorsed by the publisher.
