# Supplementary material for: IL-18/IL-18R Signaling Is Dispensable for ILC Development But Constrains the Growth of ILCP/ILCs
Source: Front Immunol. 2022 Jul 8;13:923424. doi: 10.3389/fimmu.2022.923424 (PMC9304618; doi:10.3389/fimmu.2022.923424)
Supplement: Supplementary file 1 [file DataSheet_1.docx]

**
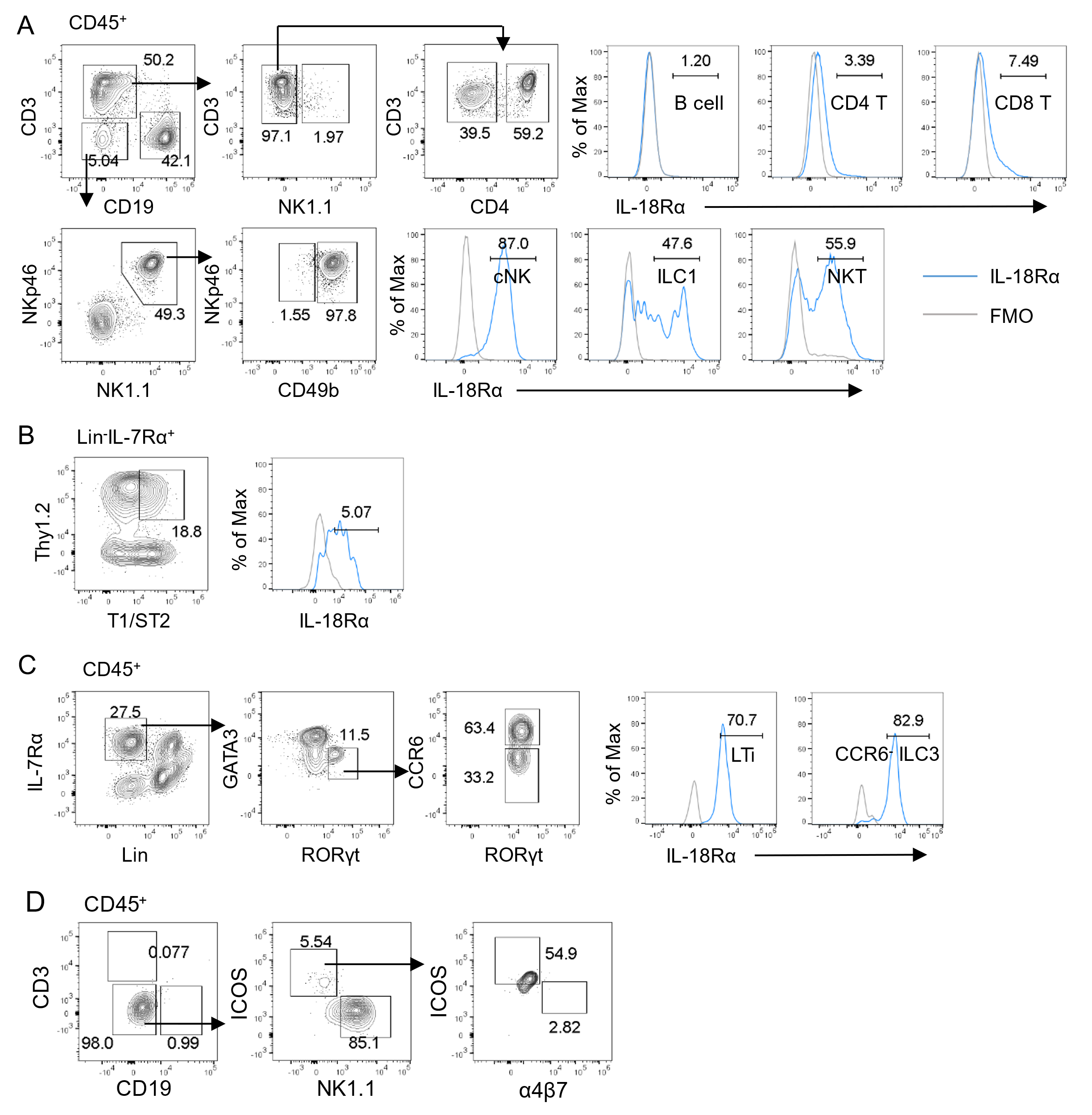
**

**Supplementary Figure 1**

**
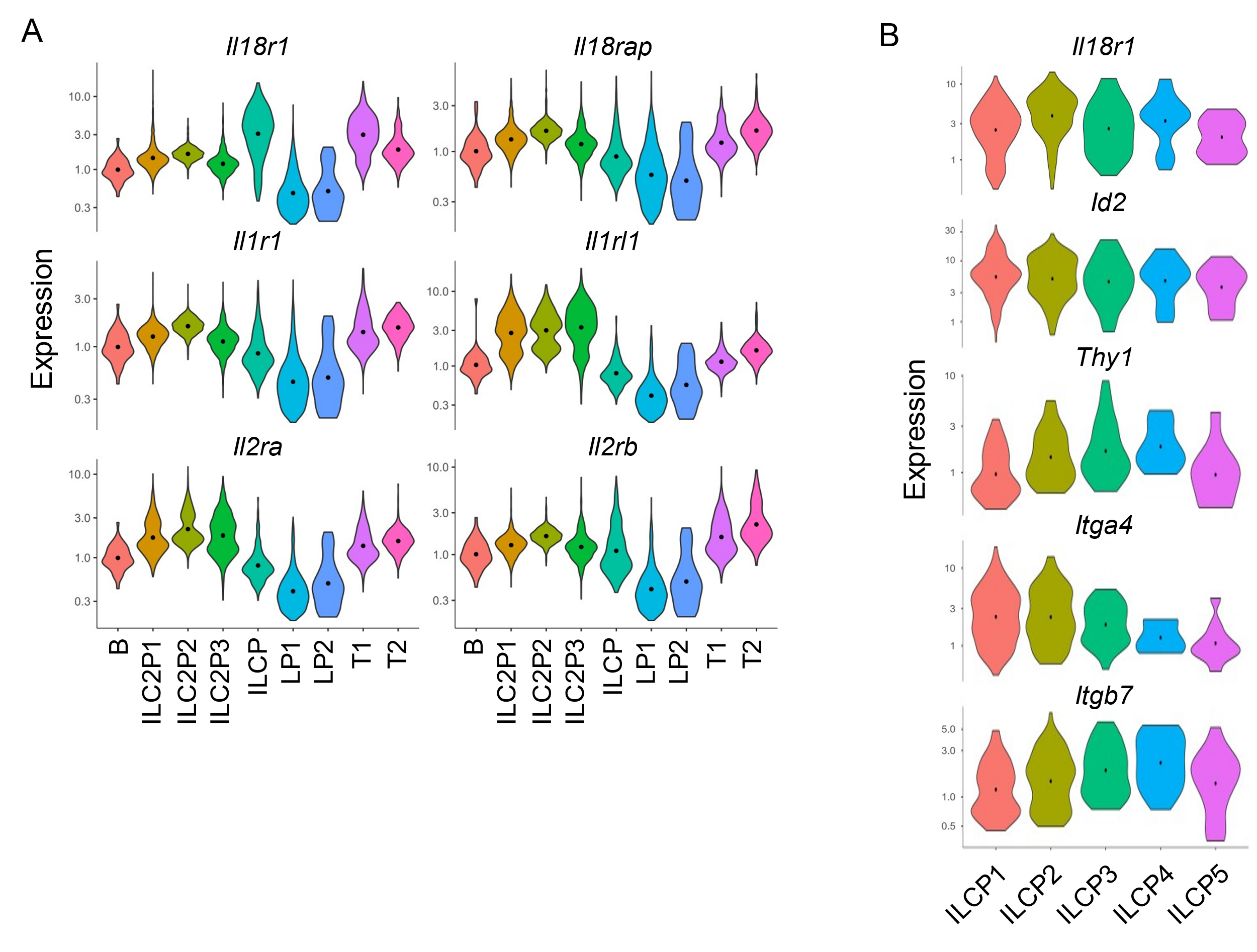
**

**Supplementary Figure 2**

**
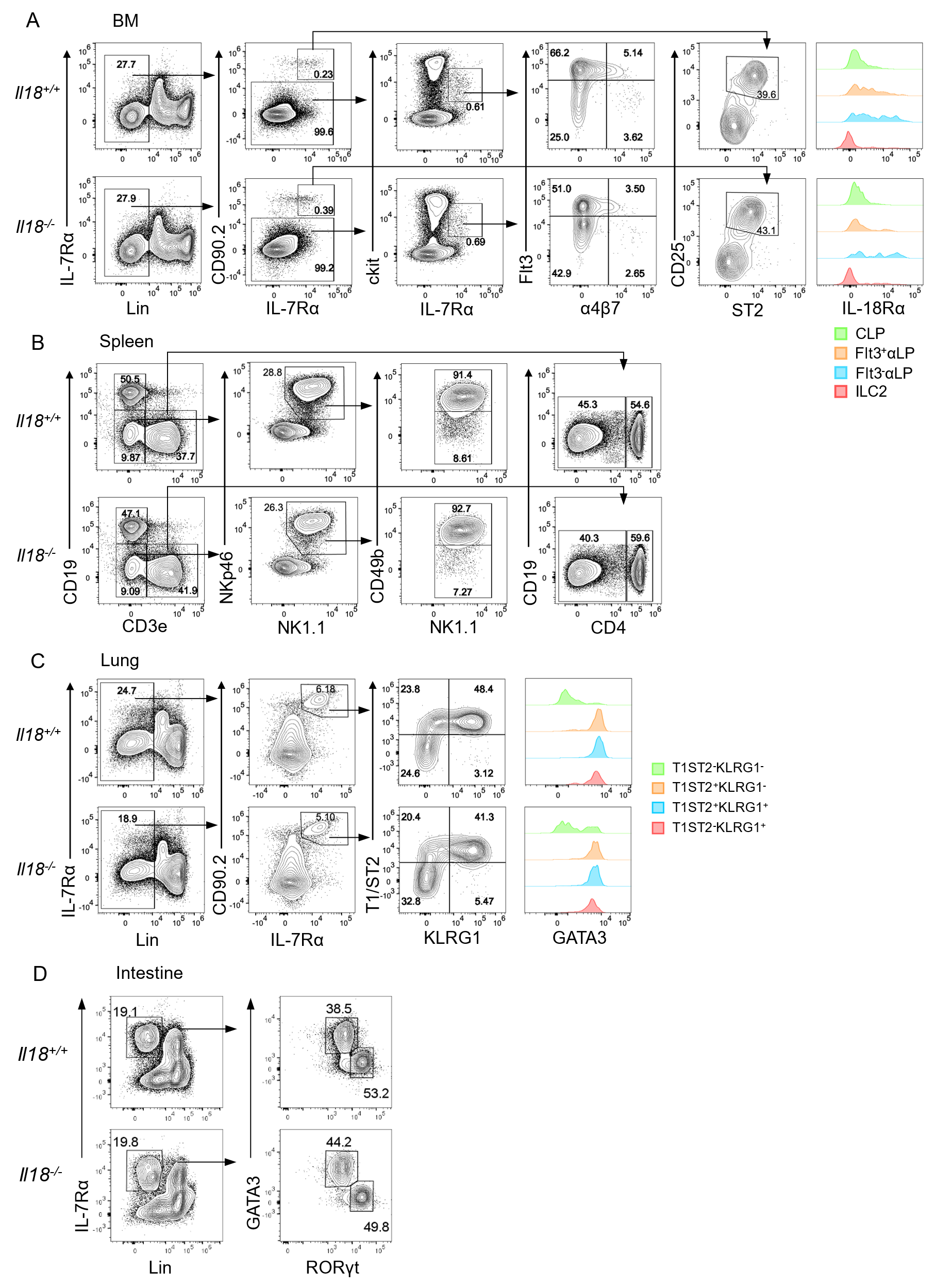
**

**Supplementary Figure 3**

**
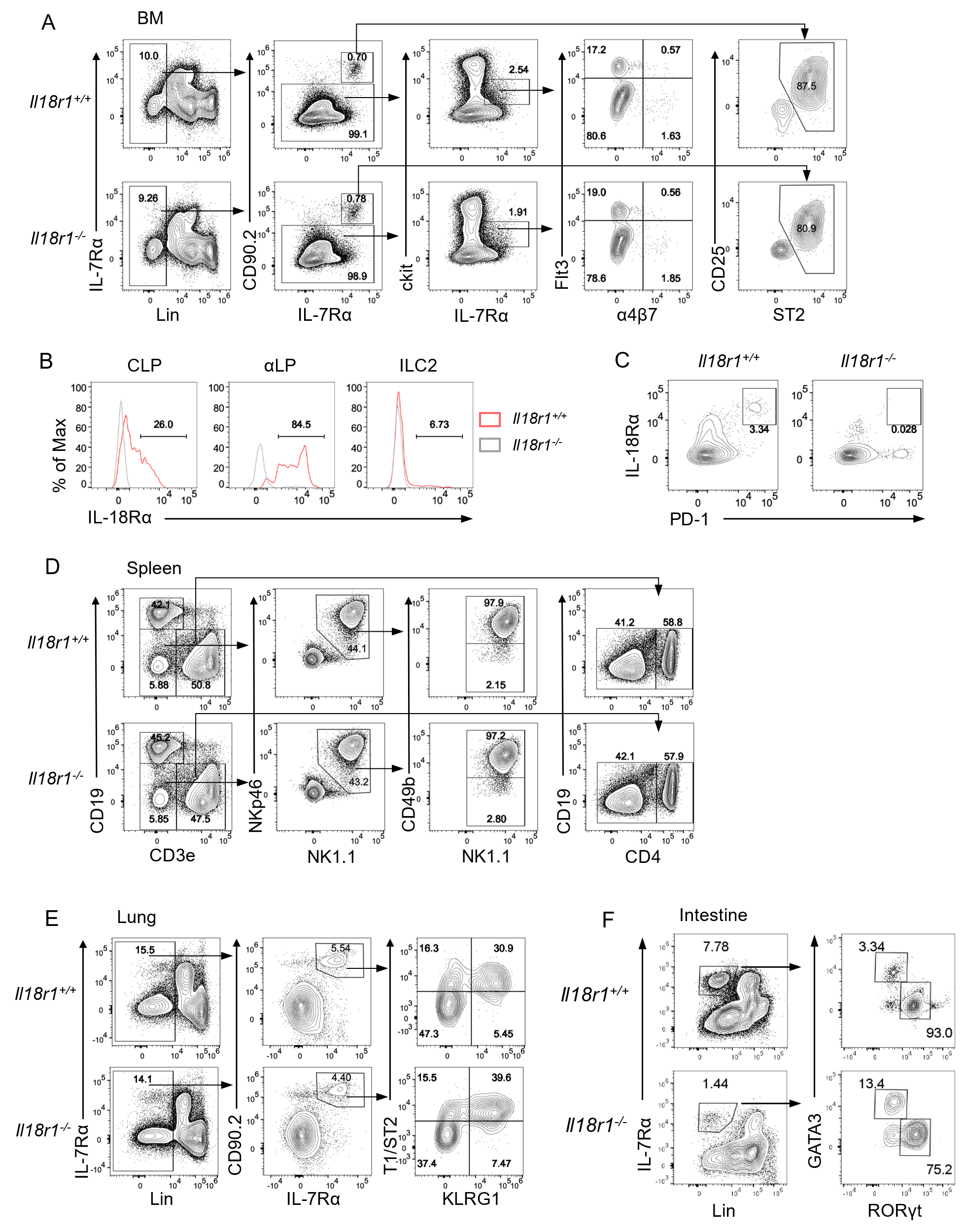
**

**Supplementary Figure 4**

**
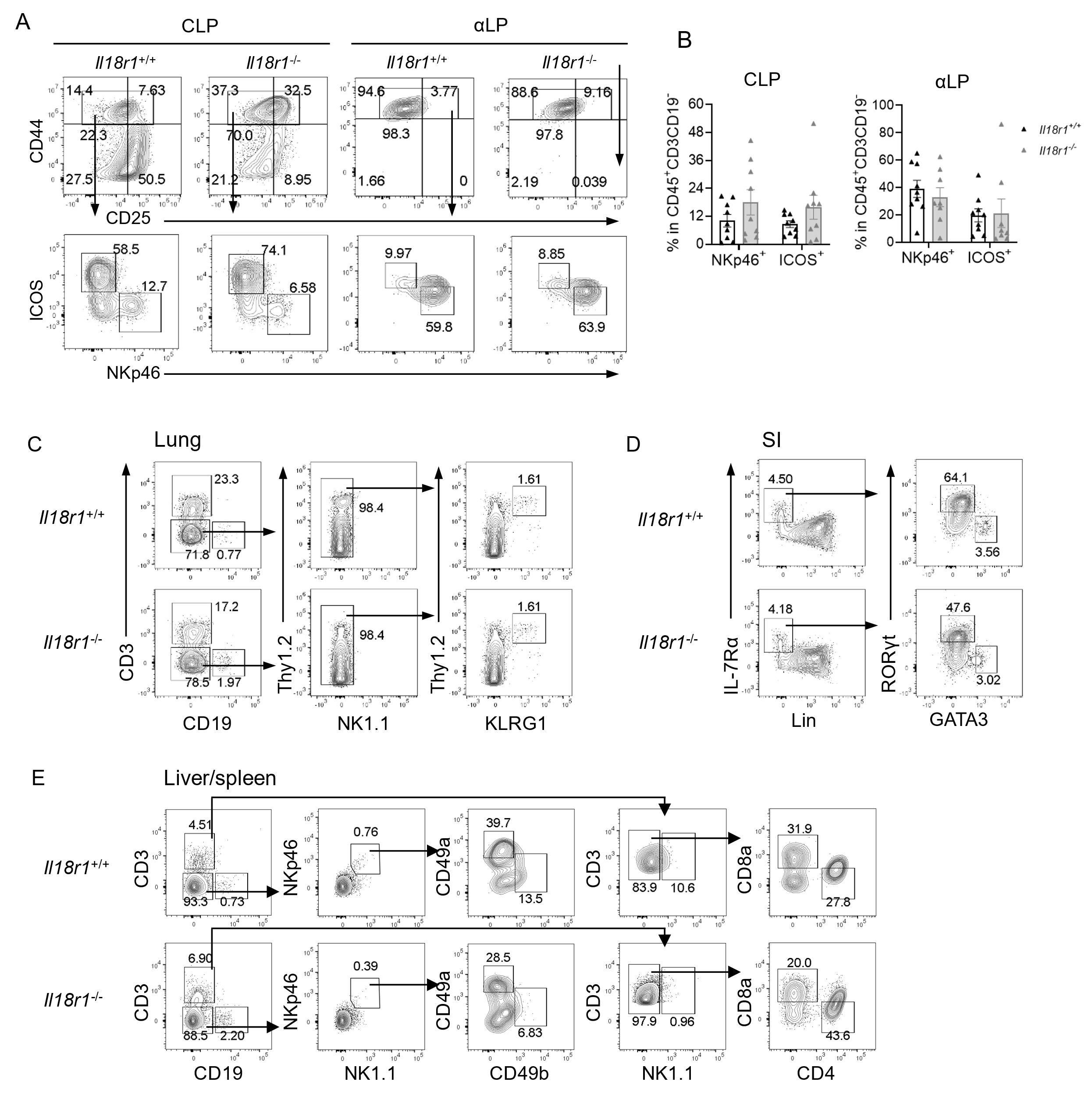
**

**Supplementary Figure 5**

**Supplementary Figure 1**

(A) Representative FACS plot showing the gating strategy of B cells (CD19^+^CD3e^-^), CD4^+^ T cells (CD19^-^CD3e^+^NK1.1^-^CD4^+^), CD8^+^ T cells (CD19^-^CD3e^+^NK1.1^-^CD4^-^), NKT cells (CD19^-^CD3e^+^NK1.1^+^), cNK cells (CD19^-^CD3e^-^NK1.1^+^NKp46^+^CD49b^+^) and ILC1 (CD19^-^CD3e^-^NK1.1^+^NKp46^+^CD49b^-^) in spleen and liver. The histograms indicate the IL-18Rα expression in these cell populations. (B) Gating strategy of ILC2 (Lin^-^CD45^+^IL-7Rα^+^Thy1.2^+^ST2^+^) in lung, the histogram shows the IL-18Rα expression in ILC2. (C) Gating strategy of CCR6^-^ ILC3 (Lin^-^IL-7Rα^+^RORγt^+^CCR6^-^) and LTi cells (Lin^-^IL-7Rα^+^RORγt^+^CCR6^+^) in intestine. The histograms indicate the IL-18Rα expression in these cell populations. Data are representative of three or more independent experiments with three or more mice in each group. (D) Representative FACS plot showing the progenies of one clone. NK1.1&ILC1 cells were defined as CD3^-^CD19^-^NK1.1^+^ICOS^-^, ILC2 were defined as CD3^-^CD19^-^NK1.1^+^ICOS^+^α4β7^-^, ILC3 were defined as CD3^-^CD19^-^NK1.1^+^ICOS^+^α4β7^+^.

**Supplementary Figure 2**

(A) Violin plots showing the expression levels of indicated cell surface receptor genes *Il18r1, Il18rap, Il1r1, Il1rl1, Il2ra, Il2rb* across 9 clusters. (B) Violin plots showing the expression levels of indicated genes across ILCP1-5.

**Supplementary Figure 3**

(A) Representative FACS plot showing BM CLP, aLP and ILC2 in *Il18^+/+^* and *Il18^-/-^* mice. (B-D) Representative FACS plot showing T, B cells, NK cells and ILC1 in spleen, ILC2 in lung, ILC2 and ILC3 in intestine from *Il18^+/+^* and *Il18^-/-^* mice.

**Supplementary Figure 4**(A) Representative FACS plot showing BM CLP, aLP and ILC2 in *Il18r1^+/+^* and *Il18r1^-/-^* mice. (B) IL-18Rα expression in BM progenitors CLP, αLP and ILC2 of *I**l18r1^+/+^* and *Il18r1^-/-^* mice. (B) Co-expression of PD-1 and IL-18Rα on BM Lin^-^IL-7Rα^+^IL-18Rα^+^ cells. (D-F) Representative FACS plot showing T, B cells, NK cells and ILC1 in spleen, ILC2 in lung, ILC2 and ILC3 in intestine from *Il18r1^+/+^* and *Il18r1^-/-^* mice.

**Supplementary Figure 5**

(A) Representative FACS plot showing *in vitro* differentiated ILCs from *Il18r1^+/+^* and *Il18r1^-/-^* CLP and αLP on OP9-DL1. (B) Percentage of NKp46^+^ NK/ILC1 and ICOS^+^ ILC2 generated from *Il18r1^+/+^* and *Il18r1^-/-^* CLP and αLP cells. Data are representative of three or more independent experiments with three or more mice in each group. The data were presented as mean±SEM, analyzed by two-tailed Student’s test. (C-E) Representative FACS plot showing the reconstitution of ILCs 5 weeks post-transplantation of *Il18r1^+/+^* or *Il18r1^-/-^* CLPs into *Rag2^-/-^Il2rγ^-/-^* mice: ILC2 (CD19^-^CD3^-^NK1.1^-^Thy1.2^+^KLRG1^+^) from lung (C), ILC2 (Lin^-^IL-7Rα^+^GATA3^+^RORγt^-^) and ILC3 (Lin^-^IL-7Rα^+^GATA3^-^RORγt^+^) from small intestine (D), B cells (CD19^+^CD3^-^), CD4^+^ T cells (CD19^-^CD3^+^NK1.1^-^CD4^+^CD8a^-^), CD8^+^ T cells (CD19^-^CD3^+^NK1.1^-^CD4^-^CD8a^+^) and cNK (CD19^-^CD3^-^NK1.1^+^NKp46^+^CD49a^-^CD49b^+^) cells ILC1 (CD19^-^CD3^-^NK1.1^+^NKp46^+^CD49a^-^CD49b^+^) from liver and spleen (E).

**Supplementary Table 1**

**List of antibodies used in this study**

| **Antibody** | **Clone** | **Conjugated Fluorochrome** | **Supplier** |
| --- | --- | --- | --- |
| α4β7 | DATK32 | APC | Invitrogen |
| CCR6 | 140706 | BV650 | BD OptiBuild |
| CD3 | 17A2 | APC-Cy7 | TONBO |
| CD3e | 145-2C11 | FITC | BD Pharmingen |
| CD4 | GK1.5 | Alexa Fluor 700 | TONBO |
| CD8a | 53-6.7 | violetFluor | TONBO |
| CD19 | ebio1D3 | Super Bright 645 | Invitrogen |
| CD19 | ebio1D3 | PE | Invitrogen |
| CD25 | PC61.5 | Super Bright 645 | Invitrogen |
| CD25 | PC61.5 | Alexa Fluor 700 | Invitrogen |
| CD44 | IM7 | PE-Cy7 | TONBO |
| CD45 | 30-F11 | BV605 | BD Horizon |
| CD45 | 30-F11 | APC | BD Pharmingen |
| CD45 | 30-F11 | APC-Cy7 | BD Pharmingen |
| CD49a | Ha31/8 | PE | BD Pharmingen |
| CD49b | DX5 | FITC | Invitrogen |
| ckit | 2B8 | PerCP-Cy5.5 | BD Pharmingen |
| ckit | ACK2 | APC-eFluor 780 | Invitrogen |
| Eomes | Dan11mag | eFluor 450 | Invitrogen |
| Flt3 | A2F10 | PE | Invitrogen |
| Gata3 | TWAJ | PerCP-eFluor 710 | Invitrogen |
| Gata3 | L50-8233 | Alexa Fluor 647 | BD Pharmingen |
| Ghost Dye Violet 510 |  | BV510 | TONBO |
| ICOS | 7E.17G9 | BV421 | BD Pharmingen |
| IL-7Rα | A7R34 | Super Bright 436 | Invitrogen |
| IL-7Rα | A7R34 | PE-Cy7 | Invitrogen |
| IL-7Rα | A7R34 | PE-Cy5 | Invitrogen |
| IL-18Rα | P3TUNYA | eFluor 450 | Invitrogen |
| IL-18Rα | P3TUNYA | PE | Invitrogen |
| IL-18Rα | P3TUNYA | PerCP-eFluor 710 | Invitrogen |
| Ki67 | soIA15 | APC-eFluor 780 | Invitrogen |
| KLRG1 | 2F1 | PE | BD Pharmingen |
| NK1.1 | PK136 | PerCP-Cy5.5 | TONBO |
| NK1.1 | PK136 | redFluor 710 | TONBO |
| NKp46 | 29A1.4 | eFluor 660 | Invitrogen |
| PD-1 | RMP1-30 | PE | Invitrogen |
| PD-1 | RMP1-30 | PE-Dazzle 594 | Biolegend |
| PLZF | R17-809 | PE | BD Pharmingen |
| RORγt | AFKJS-9 | PE | Invitrogen |
| RORγt | AFKJS-9 | APC | Invitrogen |
| Sca1 | D7 | PE-Cy7 | BD Pharmingen |
| ST2 | DJ8 | FITC | MD Biosciences |
| ST2 | RMST2-2 | Alexa Fluor 488 | Invitrogen |
| Streptavidin |  | Super Bright 780 | Invitrogen |
| Streptavidin |  | PerCP-eFluor 710 | Invitrogen |
| T-bet | eBio4B10 | PerCP-Cy5.5 | Invitrogen |
| Thy1.2 | 53-2.1 | Super Bright 645 | Invitrogen |
| Annexin V |  |  | BD Pharmingen |
| B220 | RA3-6B2 | biotin | Invitrogen |
| CD19 | 1D3 | biotin | TONBO |
| CD3e | 145-2C11 | biotin | TONBO |
| CD11b | M1/70 | biotin | TONBO |
| Ly6G/Ly6C | RB6-8C5 | biotin | BD Pharmingen |
| Ter119 | TER-119 | biotin | Invitrogen |
| CD11c | N418 | biotin | Invitrogen |
| TCRβ | H57-597 | biotin | BD Pharmingen |
| γδTCR | GL-3 | biotin | Invitrogen |
| NK1.1 | PK136 | biotin | TONBO |
| CD16/CD32 | 2.4G2 |  | TONBO |
